# P-1003. Group B Streptococcus Colonization in Pregnant Women and Their Infants: Prevalence of Colonization, Vertical Transmission, and Serotype Dynamics

**DOI:** 10.1093/ofid/ofae631.1193

**Published:** 2025-01-29

**Authors:** Byung Ok Kwak, Dong Hyun Kim

**Affiliations:** Kangnam Sacred Heart Hospital, Seoul, Seoul-t'ukpyolsi, Republic of Korea; Inha University School of Medicine, Incheon, Inch'on-jikhalsi, Republic of Korea

## Abstract

**Background:**

Group B Streptococcus (GBS) is a leading cause of neonatal mortality and morbidity worldwide. Despite historically lower colonization rates among pregnant Korean women compared to those in Western countries, recent studies indicate a rising trend in GBS colonization rates and serotype changes. This study aimed to investigate GBS colonization rates, vertical transmission, and serotype distribution among pregnant women and their infants in Korea.

**Methods:**

A prospective, mother-infant paired cohort study was conducted from January 2020 to November 2023. Vaginal and rectal swabs were obtained from pregnant women, while gastric aspirate and nasopharyngeal swabs were collected from infants immediately after birth, and nasopharyngeal and rectal swabs were obtained at one and two months of age. Serotyping and multilocus sequence typing (MLST) of GBS isolates were performed.

**Results:**

Among 427 pregnant women, 67 (15.7%) tested positive for GBS colonization. Serotype III (24%), followed by V (22.4%), VIII (19.0%), and Ib (17.2%), were the predominant serotypes, with the most common sequence types (ST) being ST2, ST19, and ST10. Vertical transmission was observed in one infant at birth, presenting with serotype V. GBS colonization was identified in four infants at one month of age, with serotypes Ia and VIII. Of these, serotype Ia persisted in two infants, while serotype III was newly detected in one infant at two months of age.

**Conclusion:**

The increasing trend in GBS colonization among pregnant Korean women highlights the need for continuous monitoring. GBS colonization was observed in infants at birth and up to two months of age, emphasizing the importance of ongoing surveillance of GBS colonization rates and serotype distribution to establish guidelines and preventive strategies in Korea.

**Disclosures:**

**All Authors**: No reported disclosures

